# A PCR-independent, annealing-free cloning method for the insertion of short DNA fragments: A simplified method for short DNA cloning

**DOI:** 10.3724/abbs.2024088

**Published:** 2024-11-26

**Authors:** Linbo Li, Jin Yan, Yuan Qi, Zhenglong Xiang, Na Jiang, Tongkang Yuan, Zhenyi Wang, Yuan Wang, Huaizhe Zhan, Shiyi Liu, Li Zhao, Jing Xu, Xiaowei Lei, Yuxuan Liu, Gui Wang, Jiayang Xie, Zhenming Guo, Chunhai Cai, Shan Bian

**Affiliations:** 1 Institute for Regenerative Medicine State Key Laboratory of Cardiology and Medical Innovation Center Shanghai East Hospital Frontier Science Center for Stem Cell Research School of Life Sciences and Technology Tongji University Shanghai 200092 China; 2 Department of Biology Brandeis University Waltham MA 02453 USA; 3 National Health Commission Key Laboratory of Birth Defect Research and Prevention Hunan Provincial Maternal and Child Health Care Hospital University of South China Changsha 410008 China; 4 China Regional Research Centre International Centre for Genetic Engineering and Biotechnology Taizhou 212200 China

Since two major biotechnological advances, the discovery of restriction endonucleases in the 1970s [1] and the invention of the polymerase chain reaction (PCR) technique in the 1980s [2], molecular cloning has become a fundamental laboratory technique in life sciences and biomedical research. This technique allows the generation of recombinant DNA molecules from selected DNA fragments and has become an extremely powerful tool for DNA manipulation [3]. Traditionally, molecular cloning involves joining linearized vectors and DNA fragments, which may have blunt ends or complementary sticky ends generated by restriction enzymes or by annealing single-stranded oligonucleotides (oligos), using T4 DNA ligase [4]. The traditional cloning procedure involves multiple steps, such as DNA purification, endonuclease digestion or annealing of single-stranded oligos, ligation, and transformation, all of which can be time-consuming. To simplify the classical molecular cloning process and increase cloning efficiency, scientists have developed several alternative cloning approaches, including endonuclease-independent TOPO cloning, ligation-independent T4 DNA polymerase cloning, overlap extension PCR cloning, recombination-mediated Gateway cloning, and Gibson assembly. Each method offers distinct advantages and is tailored to specific cloning needs, providing valuable tools for genetic engineering and molecular biology research [ 5‒ 7]. Although these methods significantly improve the efficiency of cloning relatively long DNA constructs, cloning short DNA fragments remains a time-consuming and challenging task. This underscores the critical need for innovative approaches that could specifically improve the efficiency and reduce the time required for cloning short DNA fragments.

Cloning short DNA fragments, such as short hairpin RNA (shRNA) for gene knockdown [8] or single guide RNA (sgRNA) for CRISPR/Cas9-mediated genome editing [9], is broadly used in biological and biomedical laboratories on a daily basis [10]. Typically, an annealing process is required to insert these short DNA fragments into vectors. This process involves heating single-stranded complementary sense and antisense oligos to break nonspecific hydrogen bonds, followed by slow cooling to facilitate the formation of double-stranded DNA with sticky ends. The resulting double-stranded DNA with sticky ends is then ligated into linearized vectors generated by restriction enzymes. Alternatively, short DNA fragments can be cloned using PCR-generated sequences and a Gibson assembly-based approach. However, both the annealing step and the PCR process are time-consuming and often require more than 2 h. Therefore, any optimization or novel methods that can bypass the annealing step or speed up the PCR process would significantly reduce the time required to clone short DNA fragments.

In our laboratory, a preliminary observation was made during the cloning of sgRNAs, where it was observed that two nonmatching oligos were successfully cloned and inserted into the vector. This unexpected result suggested the possibility of cloning short DNA fragments with a single oligo, using T4 DNA ligase for the ligation step and relying on host cell DNA polymerase to synthesize the complementary strand. Motivated by this hypothesis and to optimize the cloning technique for short DNA fragments, we initiated a series of experiments. Considering the potential role of host cell DNA polymerase in synthesizing the complementary strand after ligation of the template single oligo into the vector, we sought to improve cloning efficiency by adding additional T4 DNA polymerase. Since the buffers for T4 DNA ligase and T4 DNA polymerase are compatible, with each enzyme retaining 75%‒100% of its activity in the other reaction buffer, we added T4 DNA polymerase and dNTPs to the ligation mixture using either T4 DNA ligase buffer or T4 DNA polymerase buffer.

In our experimental design ( Figure 1A), we tested three setups: a sense oligo (oligo No. 001; Supplementary Table S1) alone, an antisense oligo (oligo No. 002; Supplementary Table S1) alone, and a combination of both sense and antisense oligos (oligo No. 001/002) mixed with the linearized pX458 vector. Each of these mixtures was then subjected to different treatments. They were supplemented with T4 DNA ligase alone in T4 DNA ligase buffer, T4 DNA ligase and T4 DNA polymerase (with dNTPs) in T4 DNA ligase buffer, or T4 DNA ligase and T4 DNA polymerase (with dNTPs) in T4 DNA polymerase buffer. All the samples were then incubated for one hour at 16°C. As negative controls, mixtures of oligos and linear vectors without either ligase or polymerase were also incubated under the same conditions prior to transformation. No colonies were observed from the negative controls ( Figure 1B). Surprisingly, when T4 DNA polymerase was added to the reaction mixture, almost no colony growth occurred, regardless of whether the mixtures contained a single oligo or paired oligos ( Supplementary Figure S1A). In contrast, hundreds of colonies were observed when only T4 DNA ligase was used in the ligation system. Sequencing analysis of colonies randomly selected from the sense oligo alone, antisense oligo alone, and paired oligo setups showed that ligation with paired oligos achieved the highest cloning efficiency, with 100% accuracy confirmed by sequencing. Ligation with either the sense or antisense oligo alone also achieved an 80% success rate ( Figure 1C). These results suggest that the direct ligation of single or paired oligos with T4 DNA ligase, without the annealing step, is sufficient for the efficient insertion of short DNA fragments into vectors.

**Figure 1:**
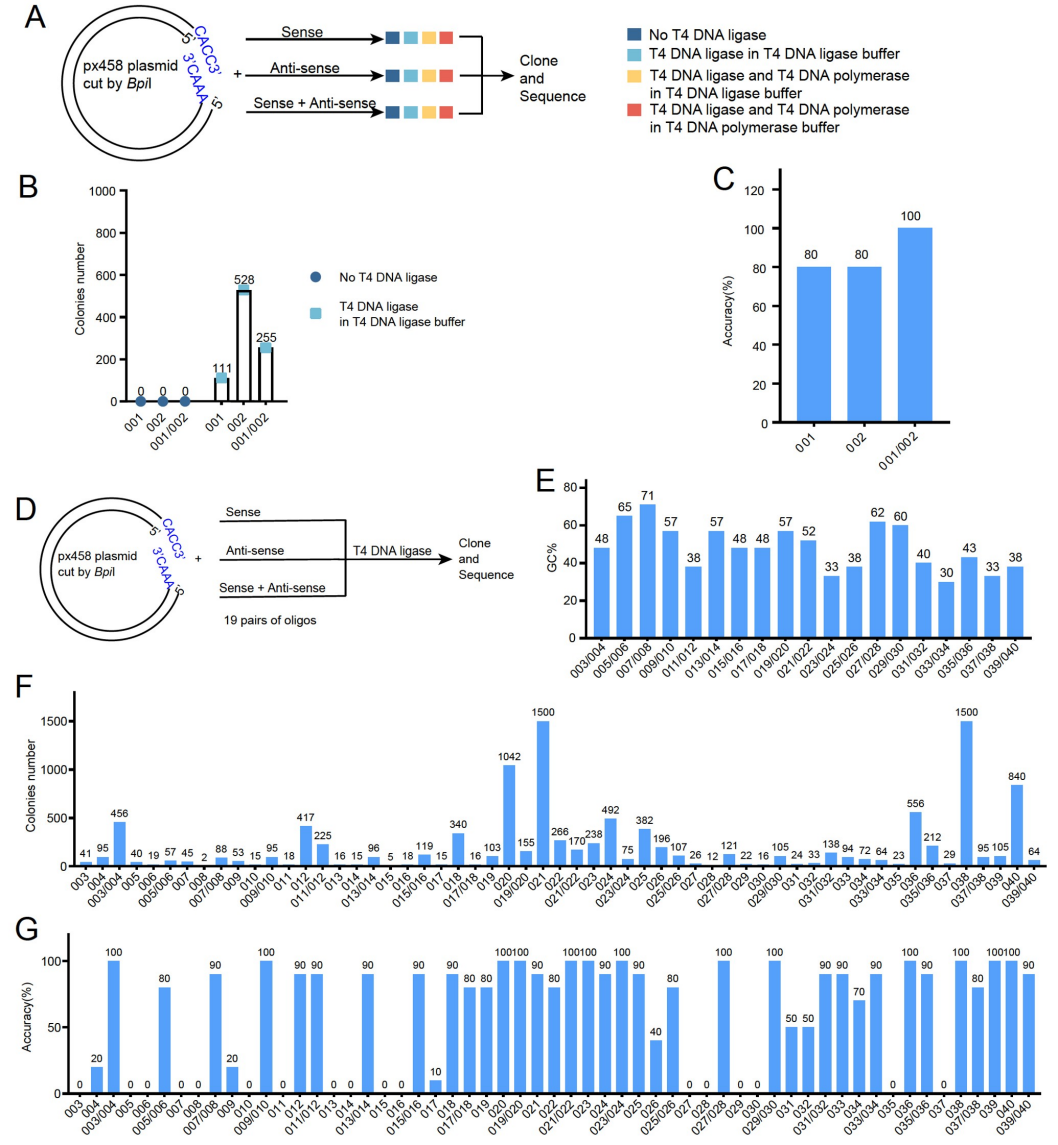
Figure 1 Paired single-stranded complementary oligos with T4 DNA ligase results in highly efficient cloning (A) The schematic illustrates the experimental design for testing the cloning of short DNA fragments using different ligation systems: sense oligo alone, antisense oligo alone, paired sense and antisense oligos without ligase (blue), T4 ligase in ligase buffer (light blue), T4 ligase and polymerase in ligase buffer (light orange), or T4 ligase and polymerase in polymerase buffer (orange). (B) The graph displays the colony numbers for each cloning condition. (C) Sequencing results indicated that the percentages of positive colonies were 80% for the sense oligo (oligo No. 001), 80% for the antisense oligo (oligo No. 002), and 100% for the paired sense and antisense oligos (oligo No. 001/002) from the cloning conditions with T4 DNA ligase in ligase buffer. (D) The schematic illustrates the experimental design for cloning 19 pairs of sgRNA oligos into the pX458 vector. (E) The GC percentage of the 19 pairs of sgRNA oligos (oligo No. 003-040). (F) The colony numbers for different cloning conditions using the sense oligo alone, antisense oligo alone, and paired oligos from the 19 pairs of sgRNA oligos (oligo No. 003-040). (G) Sequencing results showing the percentages of positive colonies for different cloning conditions using the sense oligo alone, antisense oligo alone, and paired oligos from the 19 pairs of sgRNA oligos (oligo No. 003-040).

Next, we extended our investigation to determine whether the efficacy of this cloning approach is sequence dependent. For this purpose, we randomly selected an additional 19 pairs of oligos (oligos No. 003-040; Supplementary Table S1) designed for cloning sgRNAs targeting different genes with different sequences and GC contents (ranging from 30% to 71%; Figure 1D,E). We evaluated the cloning efficiency using the direct ligation method for each of the following scenarios: the sense oligo alone, the antisense oligo alone, and the paired sense and antisense oligos. Our results showed variability in the number of colonies among the different single-stranded and paired oligos ( Figure 1F). However, sequencing analysis of 10 randomly selected colonies from each condition showed that ligation with paired oligos-across different sequences and GC content-consistently exhibited high cloning efficiency and accuracy. The correct insertion of DNA fragments was detected in more than 80% of the colonies tested by sequencing ( Figure 1G). While some single-stranded oligos also achieved high cloning efficiencies, there were numerous instances where the DNA fragments did not correctly insert into the vector when they were cloned with single-stranded oligos ( Figure 1G). This suggests that although direct ligation can be successfully applied to a variety of sequences and GC contents when paired oligos are used, the efficiency may vary when single oligos are used.

The cloning and sequencing data consistently demonstrated reliable cloning efficiency from the direct ligation of paired oligos. Next, we aimed to determine whether this method depends on sticky ends. For this investigation, we selected the pGEM-T easy vector, which provides multiple cloning sites to create different sticky ends or blunt ends. We chose eight restriction enzyme cutting sites ( *Nco*I, *Spe*I, *Sal*I, *Nde*I, *Sac*I, *Mlu*I, *Eco*RI, and *Eco*RV) and tested six combinations of sticky ends ( *Nco*I/ *Spe*I, *Nco*I/ *Sal*I, *Nco*I/ *Nde*I, *Nco*I/ *Sac*I, *Nco*I/ *Mlu*I, and *Eco*RI/ *Eco*RI) as well as one blunt end generated with *Eco*RV ( Figure 2A). Initially, we examined the self-ligation efficiency of these six sticky end combinations and the *Eco*RV-generated blunt end. The results indicated that *Eco*RI/ *Eco*RI had the highest self-ligation efficiency, while combinations of *Nco*I/ *Sal*I and *Nco*I/ *Sac*I showed extremely low self-ligation efficiency ( Figure 2B). We then assessed whether different ends affect cloning efficiency by ligating the same complementary sequences (from oligo No. 039/040) with the corresponding sticky ends or the *Eco*RV-generated blunt end (oligo No. 118-127) ( Figure 2A). Interestingly, although the number of colonies formed from different combinations was similar ( Figure 2C), the cloning efficiency and the percentage of positive colonies varied significantly ( Figure 2D). The combinations with high self-ligation efficiency, including *Eco*RI/ *Eco* RI, *Nco*I/ *Mlu*I, and *Nco*I/ *Nde*I, exhibited the lowest percentage of positive colonies (0% for *Eco*RI/ *Eco*RI, 30% for *Nco*I/ *Mlu*I, 60% for *Nco*I/ *Nde*I). Additionally, very few colonies with fully complementary sequences were positive for the *Eco*RV-generated blunt end (five pairs of oligos No. 118-127; Figure 2A,D and Supplementary Figure S1B). High percentages of positive colonies were observed for the other three sticky-end combinations (90%; Figure 2D). These observations suggest that the efficiency of direct ligation-mediated short DNA cloning largely depends on sticky ends.

**Figure 2:**
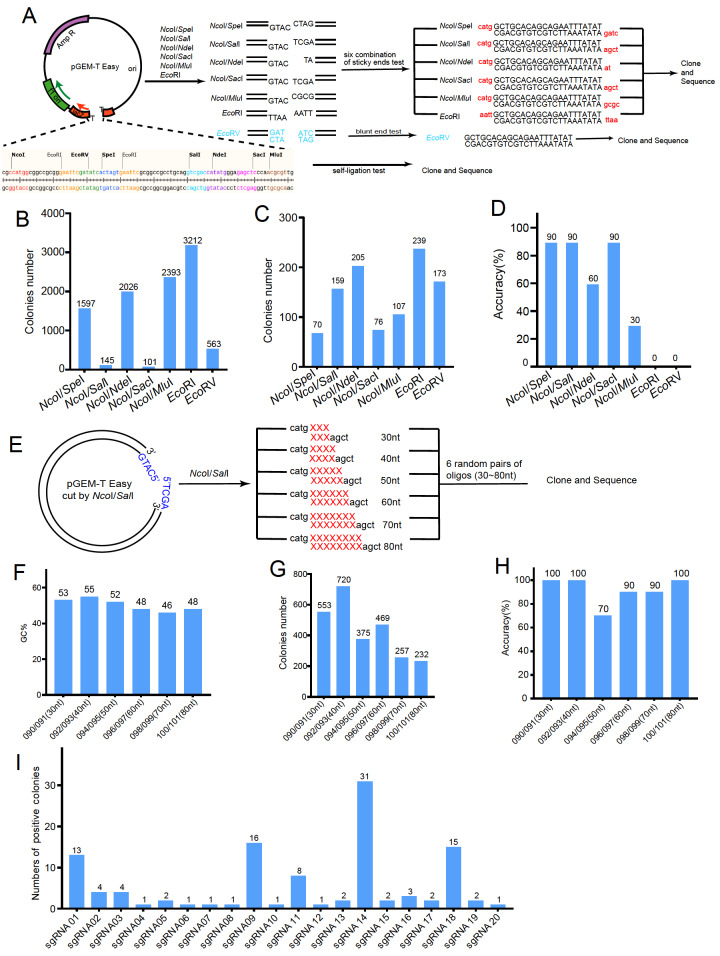
Figure 2 High cloning efficiency and accuracy with paired oligos is sticky end-dependent (A) The schematic illustrates the experimental design for testing the cloning efficiency and accuracy of paired oligos with the same complementary sequence (based on oligo No. 039/040) but different sticky ends (NcoI/SpeI, Nco I/SalI, NcoI/NdeI, Nco I/SacI, NcoI/MluI, EcoRI) or a blunt end. The pGEM-T easy vector was used to create linear vectors with different combinations of sticky ends or blunt ends generated by different restriction enzymes. (B) The colony numbers represent the self-ligation of vectors with 6 different sticky end combinations and one blunt end combination. (C) The colony numbers correspond to clones with 6 different sticky end combinations and 1 blunt end. (D) Sequencing results indicating the percentage of colonies positive for cloning with 6 different sticky end combinations and 1 blunt end. (E) The schematic illustrates the experimental design for testing the cloning efficiency of paired oligos of different lengths (30‒80 nt, oligo No. 050-061). (F) The percentage of GCs in oligos of different lengths (oligo No. 050-061). (G) Colony numbers corresponding to the cloning of oligo pairs of different lengths. (H) Sequencing results indicating the percentage of colonies positive for cloning with oligo pairs of different lengths. (I) The numbers of positive colonies of sequenced sgRNAs in the small sgRNA library.

In previous experiments, we focused on cloning 20 nt oligo pairs, which are typically used for shRNA and sgRNA cloning. For the introduction of tag sequences, such as myc-tags or flag-tags, longer DNA fragments (<90 bp) are often cloned by incorporating these sequences into primer sequences for target gene amplification. To evaluate the ability of our method to insert longer DNA fragments, we designed oligo pairs of different lengths (30, 40, 50, 60, 70, and 80 nt; Oligo No. 050-061; Supplementary Table S1) with approximately 50% GC content and sticky ends of *Nco* I/ *Sal*I, which previously showed low self-ligation and high cloning efficiency ( Figure 2F). We then performed colony counting and sequencing analysis on 10 randomly selected colonies from each condition to evaluate the performance of our method with these longer oligos ( Figure 2G). The results showed a decrease in colony counts for the longer oligo pairs compared to the shorter ones. However, the percentage of positive colonies, indicating successful cloning, remained consistently greater than 70% ( Figure 2H). These results suggest that while our method remains effective for cloning longer DNA fragments, fragment length has a significant effect on the total number of successful colonies obtained. This observation raises an interesting question about the upper limit of fragment length that our direct ligation method can efficiently handle, particularly for fragments longer than 100 nt, which are typically cloned using PCR-based methods. Further investigation is needed to explore this aspect and to determine whether our approach can be adapted for cloning even longer DNA fragments.

To further validate the reproducibility of our methodology, we performed a comprehensive evaluation focusing on three key aspects: the efficacy of different T4 DNA ligases in the ligation process, the transformation efficiency of different competent *E*. *coli* cells, and the consistency of experimental results among different researchers. First, to evaluate the performance of different T4 DNA ligases, we tested the cloning efficiency of six T4 ligases from different companies following each manufacturer’s instructions ( Supplementary Figure S1C,D). This step was critical to ensure that our cloning method was not limited to a specific brand or type of enzyme. Second, the efficiencies of the different competent strains were evaluated by transforming the ligation products into five different *E*. *coli* strains: DH5α, XL1-Blue, Mach1-T1, Top10, and DMT ( Supplementary Figure S1C,E). This diversity allowed us to determine whether our cloning method is effective across commonly used laboratory strains. Finally, to verify the consistency of our method, parallel experiments were performed by multiple researchers (randomly selecting two pairs of oligos for each individual). This approach helped to ensure that the results were reliable and reproducible, regardless of the researchers ( Supplementary Figure S1F,G). The collective results of these tests demonstrated that our method is highly reproducible, consistently yielding a high percentage (>80%) of positive colonies in tests for all three aspects of the evaluation ( Supplementary Figure S1C‒G). This consistency underscores the robustness and reliability of our cloning approach and confirms its suitability for routine use in molecular biology laboratories.

Next, we sought to further optimize the cloning efficiency of our direct ligation approach by determining the effective ratio between the short DNA fragment and the vector, as well as the optimal ligation time. To determine an efficient range of vector-to-paired oligo ratios, we performed experiments with vector-to-oligo ratios ranging from 1:1.3 to 1:130,000. The results showed that the cloning accuracy reached an impressive 100% for all tested ratios ( Supplementary Figure S1H). We also optimized the temporal parameters of the ligation process, testing a range of durations, including 5, 10, 20, 30, 40, 50, and 60 min at both 16°C and 25°C. Our method revealed that a ligation duration of 5 min is sufficient to achieve high efficiency under both temperature conditions ( Supplementary Figure S1I,J). These results suggest that our approach is highly tolerant of the ratio between vectors and short DNA fragments and that the entire cloning process can potentially be reduced to less than half an hour, significantly improving the speed and convenience of cloning short DNA fragments. The finalized procedure is described in the Supplementary Materials and Methods. We also explored the potential application of our PCR-independent, annealing-free cloning method in constructing a small sgRNA library for CRISPR-Cas9-based genetic screening. We randomly selected 20 pairs of sgRNA oligos for library construction. The coverage of the sgRNA library was subsequently analyzed by sequencing 300 randomly selected colonies. The results confirmed that all 20 sgRNAs were successfully covered in the library ( Figure 2I), demonstrating that our method is potentially suitable for constructing small sgRNA libraries. However, the number of positive colonies varied among the 20 sgRNAs, suggesting that the sequence of the DNA fragments may influence the coverage of the library when constructing a larger library, which might further impact the outcomes of genetic screening.

Our study contributes to the advancement of molecular cloning techniques by providing a highly reproducible method that simplifies the cloning process for short DNA fragments by replacing traditional annealing and PCR steps with direct ligation. In addition, we found that cloning of short DNA fragments could be achieved by mixing a single oligo with a linearized double-stranded vector. Although the cloning efficiency varied between different oligos and between sense and antisense oligos, successful cloning suggested not only that T4 DNA polymerase plays a role in this process but also that the machinery of the host cells, probably involving DNA polymerase or DNA damage repair machinery, may be involved in the cloning process. However, further investigation is needed to determine whether and how the host cell machinery plays a role in the single oligo-mediated cloning process.

## Supplementary Data

Supplementary data is available at *Acta Biochimica et Biophysica Sinica* online.
